# Neural Network for Correlated Survival Outcomes Using Frailty Model

**DOI:** 10.6339/25-jds1173

**Published:** 2025-03-26

**Authors:** Ruiwen Zhou, Kevin He, Di Wang, Lili Liu, Shujie Ma, Annie Qu, J. Philip Miller, Lei Liu

**Affiliations:** 1 Division of Biostatistics, Washington University in St. Louis, St. Louis, Missouri, USA; 2 Department of Biostatistics, University of Michigan, Ann Arbor, Michigan, USA; 3 Department of Statistics, University of California, Riverside, California, USA; 4 Department of Statistics, University of California, Irvine, California, USA

**Keywords:** correlated survival outcomes, deep learning, prediction, random effect

## Abstract

Extensive literature has been proposed for the analysis of correlated survival data. Subjects within a cluster share some common characteristics, e.g., genetic and environmental factors, so their time-to-event outcomes are correlated. The frailty model under proportional hazards assumption has been widely applied for the analysis of clustered survival outcomes. However, the prediction performance of this method can be less satisfactory when the risk factors have complicated effects, e.g., nonlinear and interactive. To deal with these issues, we propose a neural network frailty Cox model that replaces the linear risk function with the output of a feed-forward neural network. The estimation is based on quasi-likelihood using Laplace approximation. A simulation study suggests that the proposed method has the best performance compared with existing methods. The method is applied to the clustered time-to-failure prediction within the kidney transplantation facility using the national kidney transplant registry data from the U.S. Organ Procurement and Transplantation Network. All computer programs are available at https://github.com/rivenzhou/deep_learning_clustered.

## 1 Introduction

Survival models have been extensively developed in medical research to make inferences and predictions on failure times. The Cox proportional hazards model is the most commonly used regression model for survival outcomes. In the conventional Cox model, the survival outcomes from different observational units are assumed to be independent, given observed covariates.

However, dependence among survival outcomes is likely to occur. To account for within-cluster dependency, extensive literature has been published on frailty models, where the survival outcomes are assumed to be independent conditional on an unobserved frailty (random effect). In the Cox proportional hazards frailty model, the frailty or random effect is assumed to follow a probability distribution (Balan and Putter (2020)). To illustrate, Paik et al. (1994), Shih and Louis (1995), and Hens et al. (2009) assumed the frailty follows a gamma distribution, while Ripatti and Palmgren (2000) considered the log-normal frailty distribution. Other examples include the power-variance-function (PVF) family, where the marginal distribution of survival outcomes can be obtained in a closed form. Besides the frailty models, the stratified Cox model is a popular tool for clustered survival outcomes because of its simplicity in computation and interpretation. However, according to Glidden and Vittinghoff (2004), the stratified Cox model discards between-cluster comparison information, leading to inefficient estimation. This issue becomes particularly pronounced when dealing with a large number of strata or clusters, such as in the correlated survival outcomes observed in the motivating kidney transplant study.

Our motivating example is the kidney transplant registry data from the U.S. Organ Procurement and Transplantation Network (OPTN: https://optn.transplant.hrsa.gov/data/). The dataset includes the incidence of graft failure or death following transplantation for each patient across multiple kidney transplant centers. Patients from the same transplant center may receive treatments under the same protocol, adhere to uniform center policies, or be influenced by the same local environmental factors. Such commonalities may result in similar health outcomes for patients at the same transplant center. Ignoring such associations leads to inefficiency and bias in predicting the time-to-event. Furthermore, the impact of myriad of variables from both donors and recipients on the survival outcome may be complicated. It is of great interest to predict the patients’ survival outcome based on these predictors while accounting for the correlation structure within each cluster.

Recently, deep learning methods have surged as effective tools for prediction. Fan et al. (2021) discussed the theoretical foundations of deep neural networks and explained their practical and theoretical benefits over traditional statistical methods by applying depth, overparameterization, and training techniques (e.g., stochastic gradient descent and batch normalization). Neural networks improve estimation and prediction performance because they are highly flexible and can model non-linear relationships. Besides, with multiple hidden layers structure, neural networks can learn hierarchical representations of data, potentially capturing intricate patterns and interactions. In addition, modern training techniques of neural networks offer various regularization techniques (e.g., dropout and $L_{2}$ regularization) that can help prevent overfitting. These methods, when applied to survival models, have demonstrated superior predictive abilities, especially when dealing with complex nonlinear and interactive risk effects. Works by Liao and Ahn (2016), Martinsson (2017), and Ranganath et al. (2016) introduced deep learning algorithms assuming survival outcomes adhered to the Weibull distribution. Under the semiparametric Cox proportional hazards model framework, Faraggi and Simon (1995) first adopted a feed-forward neural network. Later, Katzman et al. (2018) introduced “Deepsurv”, an algorithm that harnesses advanced deep learning methodologies while minimizing the loss function derived from the partial likelihood function. Zhong et al. (2021) proposed deep extensions of the extended hazard model, named as DeepEH, which encompassed the Cox and accelerate failure time (AFT) models. Ching et al. (2018) suggested Cox-nnet for high-throughput RNA sequencing data. Hao et al. (2018) illustrated Cox-PASNet method, which integrates high-dimensional gene expression data and clinical data on a simple neural network architecture for survival analysis to improve the biological interpretation of genes and pathways. A review on deep learning methods for survival analysis was provided by Wiegrebe et al. (2023).

Despite the considerable exploration of deep learning for survival outcomes, correlated survival outcomes remain relatively untouched. Lee et al. (2023) proposed a deep neural network based on the Gamma frailty model using the H-likelihood framework. Later, Wu et al. (2024) proposed the neural frailty machine, using frailty terms for modeling crossing hazards and injecting a domain-specific inductive bias for nonparametric hazard regression. However, they did not consider within-cluster prediction for correlated survival outcomes. In our study, we introduce a neural network aimed at predicting correlated survival times under the Cox proportional hazards model with a normal frailty. This model predicts the risk score based on covariates using a feed-forward neural network. To address computational challenges, we employ a penalized partial likelihood formulation with the Laplace approximation to define the loss function.

The rest of the paper is organized as follows. Section 2 describes the proposed deep-learning method for correlated survival outcomes. In Section 3, we undertake simulation studies to evaluate the predictive performance of our proposed method against alternative methods. Section 4 presents a data analysis of kidney post-transplant graft failure or death prediction for patients from the same transplant center using our proposed method. We summarize our method and present future directions in Section 5.

## 2 Model

### 2.1 Problem Formulation

Let $T_{ij}$ denote the event time for the j-th unit within the i-th cluster, where i=1,…,s and $j=1,\ldots ,n_{i}$. The sample size $n=\sum_{i=1}^{s}n_{i}$. We denote $C_{ij}$ as the censoring time, $U_{ij}=\text{min}\left(T_{ij},C_{ij}\right)$ as the observed time, and $\Delta_{ij}=I\left\{T_{ij}⩽C_{ij}\right\}$ as the right-censored indicator. Given frailty (or random effect) $b_{i}$, the event times are assumed independent with the conditional hazard function

$$\lambda_{ij}\left(t|b_{i}\right)=\lambda_{0}\left(t\right)\;\text{exp}\;\left(X_{ij}^{T}\beta +b_{i}\right),$$

where $X_{ij}$ is the vector of explanatory variables, $\lambda_{0}(t)$ represents the baseline hazard function. In this frailty model, only the random intercept is considered, which follows a normal distribution with mean 0 and variance θ. We can also consider more complicated forms of random effects, e.g., replacing $b_{i}$ by $Z_{ij}^{T}u_{i}$, where $u_{i}\sim N\left(0,\Sigma_{u}\right)$ is a vector of random effects and $Z_{ij}$ is the associated covariate vector.

To better describe the covariate effects, we consider a feed-forward artificial neural network (FNN) with L hidden layers. We adapt the classical FNN under Cox proportional hazards model to a deep learning method within the frailty model framework, which may lead to more accurate hazard function estimates and improved survival predictions. The covariate $X_{ij}$ has p variables, and $X_{ij}^{T}\beta$ can be replaced by a nonlinear function of the predictors $X_{ij}$ with network weights $\omega^{\left(l\right)}$ and bias $\delta^{\left(l\right)}$ through a series of nested activation function $g_{l}(\cdot )$ for layers l=0,…,L. Weights and biases are also called slope coefficients and intercepts, respectively, in statistical terms. To be specific, the $k_{0}$ nodes of the first hidden layer can be calculated through

$$\alpha_{ij}^{\left(0\right)}=g_{0}\left\{\omega^{\left(0\right)}X_{ij}+\delta^{\left(0\right)}\right\},$$

where $\omega^{\left(0\right)}$ is a $k_{0}\times p$ weight matrix, $\delta^{\left(0\right)}$ is a bias vector of length $k_{0}$, and the activation function $g_{0}(\cdot )$ is applied element-wise to its input vector. For the l-th hidden layer (l=1,…,L-1) with $k_{l}$ nodes, the layer’s output is

$$\alpha_{ij}^{\left(l\right)}=g_{l}\left\{\omega^{\left(l\right)}\alpha_{ij}^{\left(l-1\right)}+\delta^{\left(l\right)}\right\},$$

where $\omega^{\left(l\right)}$ is a $k_{l}\times k_{l-1}$ matrix and $\delta^{\left(l\right)}$ is of length $k_{l}$. Finally, when only random intercept is considered, the univariate output from the neural network is related to the proportional hazards function by

(1) $$\lambda_{ij}^{NN}\left(t|b_{i}\right)=\lambda_{0}\left(t\right)\;\text{exp}\left(\alpha_{ij}^{\left(L\right)}+b_{i}\right),$$

where $\alpha_{ij}^{\left(L\right)}=g_{L}\left(\omega^{\left(L\right)}\alpha_{ij}^{\left(L-1\right)}\right),\alpha_{ij}^{\left(L-1\right)}$ is the second to the last layer’s output, and $\omega^{\left(L\right)}$ is a $1\times k_{L-1}$ vector.

### 2.2 Penalized Partial Likelihood

The marginal likelihood for cluster i in model (1) is

$$L_{i}^{NN}\left(\lambda_{0}(t),\omega ,\delta ,\theta \right)=\int \;\prod_{j=1}^{n_{i}}\text{exp}\left[l_{ij}^{NN}\left(\lambda_{0}\left(t\right),\omega ,\delta |b_{i}\right)\right]p\left(b_{i};\theta \right)db_{i},$$

where

$$l_{ij}^{NN}\left(\lambda_{0}(t),\omega ,\delta |b_{i}\right)=\Delta_{ij}\left[\text{log}\left(\lambda_{0}(t)\right)+\alpha_{ij}^{\left(L\right)}+b_{i}\right]-\Lambda_{0}\left(t\right)\text{exp}\left(\alpha_{ij}^{\left(L\right)}+b_{i}\right),$$

and

$$p\left(b_{i};\theta \right)=\theta^{-1/2}(2\pi {)}^{-1/2}\text{exp}\left(-\frac{1}{2}{b_{i}}^{\prime }\theta^{-1}b_{i}\right).$$

The function $\Lambda_{0}(t)=\int_{0}^{t}\lambda_{0}(u)du$ is the baseline cumulative hazard function, $l_{ij}^{NN}\left(\cdot |b_{i}\right)$ denotes the log likelihood function for subject j in the i-th cluster given random effect $b_{i}$. The parameter ω represents the combined vectorization of $\omega^{\left(0\right)},\ldots ,\omega^{\left(L\right)}$ into a single column vector, δ represents the concatenation of $\delta^{\left(0\right)},\ldots ,\delta^{\left(L-1\right)}$ into a column vector. To avoid overfitting, following Mandel et al. (2023), we add $L_{2}$ penalization to the neural network parameters ω and δ, with regularization parameter γ.

The likelihood function for model (1) in cluster i with parameter regularization then becomes

(2) $${\tilde{L}}_{i}^{NN}\left(\lambda_{0}(t),\omega ,\delta ,\theta \right)=\theta^{-1/2}(2\pi {)}^{-1/2}\int \;\text{exp}\left[\sum_{j=1}^{n_{i}}\left\{\Delta_{ij}\left[\text{log}\left(\lambda_{0}(t)\right)+\alpha_{ij}^{\left(L\right)}+b_{i}\right]-\Lambda_{0}(t)\times \text{exp}\left(\alpha_{ij}^{\left(L\right)}+b_{i}\right)-\frac{1}{2}{b_{i}}^{\prime }\theta^{-1}b_{i}-\gamma \left(\omega^{T}\omega +\delta^{T}\delta \right)\right\}\right]db_{i}.$$

Under the normal distribution assumption for the frailty term, equation (2) is difficult to maximize with an integral. Following Ripatti and Palmgren (2000), we use a Laplace approximation for the integral in ${\tilde{L}}_{i}^{NN}\left(\lambda_{0}(t),\omega ,\delta ,\theta \right)$. This leads to the approximated marginal log-likelihood for cluster i,

$$\text{log}\left({\tilde{L}}_{i}^{NN}\right)=l_{i}\left(\lambda_{0}(t),\omega ,\delta ,\theta \right)\approx -\frac{1}{2}\text{log}(\theta )-\frac{1}{2}\text{log}\left|K_{i}^{\prime \prime }\left({\tilde{b}}_{i}\right)\right|-K_{i}\left({\tilde{b}}_{i}\right)-n_{i}\gamma \left(\omega^{T}\omega +\delta^{T}\delta \right),$$

where

(3) $$K_{i}\left({\tilde{b}}_{i}\right)=-\sum_{j=1}^{n_{i}}\left[\Delta_{ij}\left[\text{log}\left(\lambda_{0}(t)\right)+\alpha_{ij}^{\left(L\right)}+{\tilde{b}}_{i}\right]+\Lambda_{0}(t)\text{exp}\left(\alpha_{ij}^{\left(L\right)}+{\tilde{b}}_{i}\right)+\frac{1}{2}{\tilde{b}}_{i}^{\prime }\theta^{-1}{\tilde{b}}_{i}\right]$$

and

(4) $$K_{i}^{\prime \prime }\left({\tilde{b}}_{i}\right)=\frac{\partial^{2}K\left({\tilde{b}}_{i}\right)}{\partial^{2}{\tilde{b}}_{i}}=\sum_{j=1}^{n_{i}}\left[\Lambda_{0}(t)\text{exp}\left(\alpha_{ij}^{\left(L\right)}+{\tilde{b}}_{i}\right)+\theta^{-1}\right].$$

The parameter ${\tilde{b}}_{i}={\tilde{b}}_{i}(\omega ,\delta )$ denotes the solution to the partial derivatives of $K_{i}\left(b_{i}\right)$ with respect to $b_{i}$.

According to Lin et al. (2008); Yu and Liu (2011); Yu et al. (2013, 2014), omitting the complicated term $\text{log}\left|K_{i}^{\prime \prime }\left({\tilde{b}}_{i}\right)\right|$ in $\text{log}\left({\tilde{L}}_{i}^{NN}\right)$ has a negligible effect on the parameter estimation. Their simulation studies demonstrate that this simplification does not significantly affect the accuracy of the estimated parameters. Consequently, we have excluded this term from our likelihood approximation to streamline computation without compromising model performance. Further, for right-censored data, to avoid estimating the baseline hazard function, replacing the full likelihood in $K_{i}\left({\tilde{b}}_{i}\right)$ with a partial likelihood leads to the following penalized approximated partial log-likelihood

(5) $$pl=\sum_{i=1}^{s}pl_{i}=\sum_{i=1}^{s}\sum_{j=1}^{n_{i}}\left\{\Delta_{ij}\left[\left(\alpha_{ij}^{\left(L\right)}+b_{i}\right)-\text{log}\sum_{d,q\in R\left(t_{ij}\right)}\text{exp}\left(\alpha_{dq}^{\left(L\right)}+b_{q}\right)\right]-\frac{1}{2}b_{i}^{\prime }\theta^{-1}b_{i}\right\}-n\gamma \left(\omega^{T}\omega +\delta^{T}\delta \right),$$

where $R\left(t_{ij}\right)$ denotes indexes for subjects who are at risk at time $t_{ij}$.

**Figure 1:**
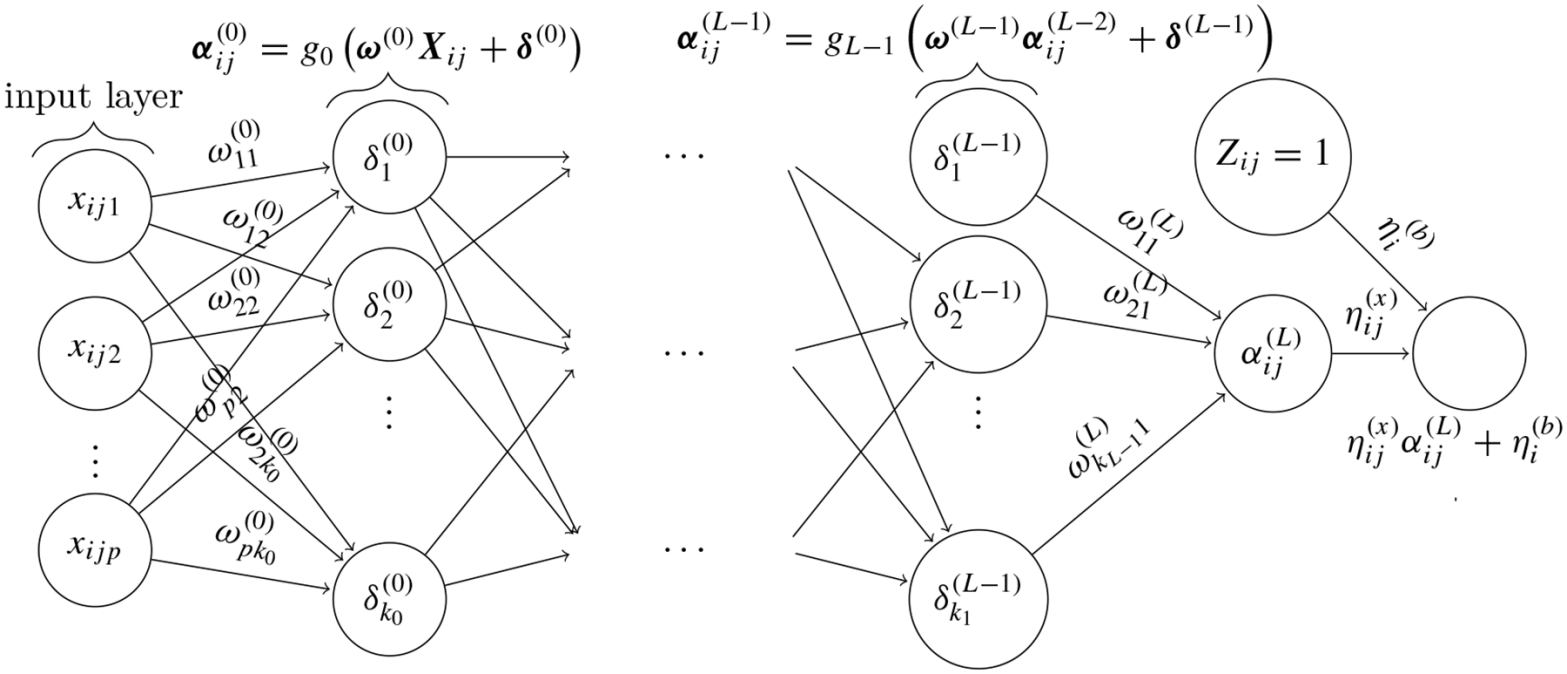
Network graph of a (L+1)-layer perceptron with p input units. The random effect intercept $b_{i}$ and indicator covariates $Z_{ij}=1$ are included in the final layer of the network.

Ripatti and Palmgren’s (2000) method estimates the fixed effects and random effects $b_{i}$ using an iterative approach, alternating between estimating equations. To be specific, in the iterative algorithm, given θ, we can estimate (ω,δ) by solving $\frac{\partial pl}{\partial \omega }=0$ and $\frac{\partial pl}{\partial \delta }=0$. Then, given the updated (ω,δ), the random effect $b_{i}$ is updated by solving $\frac{\partial pl}{\partial b_{i}}=0$. These steps are repeated until convergence. This approach, however, complicates backpropagation and imposes a substantial computational burden, as it requires multiple nested loops to iteratively update fixed and random effects. To address the computational complexity of this iterative algorithm, we propose an alternative loss function for estimating (ω,δ) and the random effect $b_{i}$,

(6) $$\text{plnn}=\sum_{i=1}^{s}\sum_{j=1}^{n_{i}}\left\{\Delta_{ij}\left[\left(\eta_{ij}^{\left(x\right)}\alpha_{ij}^{\left(L\right)}+\eta_{i}^{\left(b\right)}\right)-\text{log}\sum_{d,q\in R\left(t_{ij}\right)}\text{exp}\left(\eta_{dq}^{\left(x\right)}\alpha_{dq}^{\left(L\right)}+\eta_{q}^{\left(b\right)}\right)\right]-\frac{1}{2}\eta_{i}^{(b)\prime }\theta^{-1}\eta_{i}^{\left(b\right)}\right\}-n\gamma \left(\omega^{T}\omega +\delta^{T}\delta \right),$$

where $\alpha_{ij}^{\left(L\right)}=g_{L}\left(\omega^{\left(L\right)}\alpha_{ij}^{\left(L-1\right)}\right),\;\eta^{\left(x\right)}=\left(\eta_{11}^{\left(x\right)},\ldots ,\eta_{sc}^{\left(x\right)}\right)$, and $\eta^{\left(b\right)}=\left(\eta_{1}^{\left(b\right)},\ldots \eta_{c}^{\left(b\right)}\right)$ are weights for the final output layer. This allows the parameters $\omega ,\delta ,\eta_{ij}^{\left(x\right)},{\overset{ˆ}{b}}_{i}={\overset{ˆ}{\eta }}_{i}^{\left(b\right)}$ to be updated simultaneously during neural network backpropagation, bypassing the need for iterative updates and streamlining the estimation process. Consequently, rather than estimating the neural network parameters and the random effect $b_{i}$ iteratively, we can estimate and update $b_{i}$ in a single step using ${{\overset{ˆ}{\eta }}_{i}}^{\left(b\right)}$. Figure 1 illustrates our proposed neural network structure. In this structure, ${\overset{ˆ}{\eta }}^{\left(b\right)}$ is updated simultaneously with other model parameters $\left(\overset{ˆ}{\omega }=\left\{{\overset{ˆ}{\omega }}^{\left(0\right)},\ldots ,{\overset{ˆ}{\omega }}^{\left(L\right)}\right\},\overset{ˆ}{\delta }=\left\{{\overset{ˆ}{\delta }}^{\left(0\right)},\ldots ,{\overset{ˆ}{\delta }}^{\left(L-1\right)}\right\},{\overset{ˆ}{\eta }}^{\left(x\right)}\right)$. For a single-layer network, differentiation of the approximated partial likelihood with respect to $\eta^{\left(x\right)},\eta^{\left(b\right)},\omega ,\delta$ leads to the following quasi-score equations with $\alpha_{ij}^{\left(1\right)}=g_{1}\left(\omega^{\left(1\right)}\alpha_{ij}^{\left(0\right)}\right)$ :

(7) $$\frac{\partial \text{plnn}}{\partial \eta_{ij}^{\left(x\right)}}=\Delta_{ij}\left(\alpha_{ij}^{\left(1\right)}-\frac{\alpha_{ij}^{\left(1\right)}\text{exp}\left(\eta_{ij}^{\left(x\right)}\alpha_{ij}^{\left(1\right)}+\eta_{i}^{\left(b\right)}\right)}{\sum_{d,q\in R\left(t_{ij}\right)}\text{exp}\left(\eta_{dq}^{\left(x\right)}\alpha_{dq}^{\left(1\right)}+\eta_{q}^{\left(b\right)}\right)}\right),$$

(8) $$\frac{\partial \text{plnn}}{\partial \eta_{i}^{\left(b\right)}}=\sum_{j=1}^{n_{i}}\Delta_{ij}\left(1-\frac{\text{exp}\left(\eta_{ij}^{\left(x\right)}\alpha_{ij}^{\left(1\right)}+\eta_{i}^{\left(b\right)}\right)}{\sum_{d,q\in R\left(t_{ij}\right)}\text{exp}\left(\eta_{dq}^{\left(x\right)}\alpha_{dq}^{\left(1\right)}+\eta_{q}^{\left(b\right)}\right)}\right)-\eta_{i}^{\left(b\right)}\theta^{-1},$$

(9) $$\frac{\partial \text{plnn}}{\partial \omega_{k1}^{\left(1\right)}}=\sum_{i=1}^{s}\sum_{j=1}^{n_{i}}\Delta_{ij}\left(\eta_{ij}^{\left(x\right)}-\frac{\eta_{ij}^{\left(x\right)}\text{exp}\left(\eta_{ij}^{\left(x\right)}\alpha_{ij}^{\left(1\right)}+\eta_{i}^{\left(b\right)}\right)}{\sum_{d,q\in R\left(t_{ij}\right)}\text{exp}\left(\eta_{dq}^{\left(x\right)}\alpha_{dq}^{\left(1\right)}+\eta_{q}^{\left(b\right)}\right)}\right)\cdot g_{1}^{\prime }\left(\omega^{\left(1\right)}\alpha_{ij}^{\left(0\right)}\right)g_{0}\left(\omega_{.k}^{\left(0\right)}x_{ij}+\delta_{k}^{\left(0\right)}\right)-2n\gamma \omega_{k1}^{\left(1\right)},$$

(10) $$\frac{\partial \text{plnn}}{\partial \omega_{lk}^{\left(0\right)}}=\sum_{i=1}^{s}\sum_{j=1}^{n_{i}}\Delta_{ij}\left(\eta_{ij}^{\left(x\right)}-\frac{\eta_{ij}^{\left(x\right)}\text{exp}\left(\eta_{ij}^{\left(x\right)}\alpha_{ij}^{\left(1\right)}+\eta_{i}^{\left(b\right)}\right)}{\sum_{d,q\in R\left(t_{ij}\right)}\text{exp}\left(\eta_{dq}^{\left(x\right)}\alpha_{dq}^{\left(1\right)}+\eta_{q}^{\left(b\right)}\right)}\right)\;g_{1}^{\prime }\left(\omega^{\left(1\right)}\alpha_{ij}^{\left(0\right)}\right)\omega_{k1}^{\left(1\right)}g_{0}^{\prime }\left(\omega_{.k}^{\left(0\right)}x_{ij}+\delta_{k}^{\left(0\right)}\right)x_{ijl}-2n\gamma \omega_{lk}^{\left(0\right)},$$

(11) $$\frac{\partial \text{plnn}}{\partial \delta_{k}^{\left(0\right)}}=\sum_{i=1}^{s}\sum_{j=1}^{n_{i}}\Delta_{ij}\left(\eta_{ij}^{\left(x\right)}-\frac{\eta_{ij}^{\left(x\right)}\text{exp}\left(\eta_{ij}^{\left(x\right)}\alpha_{ij}^{\left(1\right)}+\eta_{ij}^{\left(b\right)}\right)}{\sum_{d,q\in R\left(t_{ij}\right)}\text{exp}\left(\eta_{dq}^{\left(x\right)}\alpha_{dq}^{\left(1\right)}+\eta_{q}^{\left(b\right)}\right)}\right)\cdot \;g_{1}^{\prime }\left(\omega^{\left(1\right)}\alpha_{ij}^{\left(0\right)}\right)\omega_{k1}^{\left(1\right)}g_{0}^{\prime }\left(\omega_{.k}^{\left(0\right)}x_{ij}+\delta_{k}^{\left(0\right)}\right)-2n\gamma \delta_{k}^{\left(0\right)},$$

where $\eta^{\left(x\right)}$ and $\eta^{\left(b\right)}$ are the weights for the last layer, $\omega_{k1}^{\left(1\right)}$ is the weight connecting the k-th hidden node to the univariate output $\alpha_{ij}^{\left(1\right)},\;\omega_{lk}^{\left(0\right)}$ is the weight connecting the l-th input to the k-th hidden node in the hidden layer, $\delta_{k}^{\left(0\right)}$ is the bias of the k-th hidden node in the hidden layer, and $\omega_{\cdot k}^{\left(0\right)}$ is the k-th entry of the vector $\omega^{\left(0\right)}$.

To train the neural network, we develop our code along the lines of the Deepsurv method (Katzman et al. (2018), Kvamme et al. (2019)): standardization of the continuous input, Adaptive Moment Estimation (Adam) for the gradient descent algorithm, Nesterov momentum, and learning rate schedule. We tune the hyperparameter exponential learning rate decay constant and apply inverse time decay to the learning rate at each epoch. Since the goal is prediction, we will focus on the estimation of ($\omega ,\delta ,\eta^{\left(x\right)},\eta^{\left(b\right)}$). The parameter θ is estimated by solving the estimating equation derived from penalized partial likelihood function as in Ripatti and Palmgren (2000). The baseline hazard function can be estimated with a Breslow-type estimator:

$${\hat{\Lambda }}_{0}\left(t\right)=\sum_{i,j:x_{ij}⩽\text{t}}\frac{\Delta_{ij}}{\Sigma_{d,q\in R\left(x_{ij}\right)}\text{exp}\left({\overset{ˆ}{\eta }}_{dq}^{\left(x\right)}g_{1}\left({\overset{ˆ}{\omega }}^{\left(1\right)}{\overset{ˆ}{\alpha }}_{dq}^{\left(0\right)}\right)+{\overset{ˆ}{\eta }}_{q}^{\left(b\right)}\right)}.$$

## 3 Simulation Study

We generate the data under the Cox model with shared frailty and nonlinear effects (true model). Then we compare the proposed method to (i) Deepsurv, (ii) the Cox model with only linear effects, (iii) the Cox model with linear effects and all two-way interactions, (iv) the Cox model with frailty and linear effects, (v) the Cox model with frailty, linear and all two-way interaction effects, and (vi) the Cox with fixed clustering effects. As in the real data analysis, we are interested in the within-cluster prediction; so for subjects within a cluster, we randomly assign 50% subjects to the training dataset and the other 50% subjects to the test dataset. The ReLU (Rectified Linear Unit) activation function is selected in the neural network prediction.

To evaluate the performance of the models in terms of discrimination, we adopt the concordance index (C-index), a measure of the rank correlation between predicted risk scores and observed time points (Harrell Jr et al. (1984)). If C-index = 0.5, the method is the same as a random guess. If C-index = 1, the ranking of predicted risk scores perfectly matches that of the observed death times.

The data are generated from a proportional hazards model,

$$\lambda_{ij}\left(t|b_{i}\right)=\lambda_{0}\left(t\right)\text{exp}\left(r_{ij}\right),$$

where $i=1,\ldots ,s;j=1,\ldots ,c;\lambda_{0}(t)=1$ is an exponential baseline hazard; and $r_{ij}$ is the risk score. To mimic the observations in the motivating example of kidney transplant data, we have s=200 clusters, and the sizes of these clusters are randomly drawn from a distribution defined by $n_{i}\sim \;\text{Uniform}(20,100)$. Four values for the frailty variance are used, i.e., θ=0,1.5,2.5,and3.5. We consider two scenarios for covariates $X_{ij}$. In scenario 1, we first generate five independent variables $M_{ij}={\left(M_{ij1},M_{ij2},M_{ij3},M_{ij4},M_{ij5}\right)}^{T}$ from normal distributions with mean 0 and variance 1, we then set $r_{ij}=X_{ij}^{T}\beta -3+b_{i}$. Following the setup in Katzman et al. (2018), the covariates are calculated by $X_{ij}={\left(M_{ij1}^{2},M_{ij2}^{2},M_{ij3}^{2},M_{ij4}^{2},M_{ij5}^{2}\right)}^{T}$, where no interactive covariate effects are considered here, and the parameters are $\beta ={\left(\beta_{1},\beta_{2},\beta_{3},\beta_{4},\beta_{5}\right)}^{T}=(0.5,0.5,0.5,0.5,0.5{)}^{T}$. The censoring times are generated from Uniform(0, 0.5) with around 70% of the event times independently right-censored. In scenario 2, we first generate 15 independent variables $M_{ij}=\left(M_{ij1}\right.,{\left.M_{ij2},M_{ij3},M_{ij4},M_{ij5},M_{ij6},M_{ij7},M_{ij8},M_{ij9},M_{ij10},M_{ij11},M_{ij12},M_{ij13},M_{ij14},M_{ij15}\right)}^{T}$ from normal distributions with mean (1, 1, 1, 2, 2, 3, 3, 3, 0, 0, 0, 0, 0, 0, 0) and variance 1, and then set $M_{ij8}=I\left(M_{ij8}<1\right)$ to generate a binary covariate. We generate more complicated nonlinear effects inspired by case 3 in Zhong and Wang (2023): $X_{ij}=\left\{X_{ij1},X_{ij2},X_{ij3},X_{ij4}\right\}=\left\{0.1\;\text{exp}\left(M_{ij1}(1+\right.\right.\left.\left.M_{ij2}-M_{ij3}M_{ij4}M_{ij5}\right)/2\right)\left|M_{ij5}+0.2-0.01M_{ij9}M_{ij10}\right|,M_{ij5}\left(M_{ij3}M_{ij4}-0.3\right)/\left(|2M_{ij3}M_{ij4}M_{ij6}-1+\right.\left.0.01M_{ij11}M_{ij12}|+1\right),2\;\text{sin}\left(M_{ij1}M_{ij2}M_{ij5}\right)\left|M_{ij2}M_{ij5}M_{ij6}-0.6-0.01M_{ij13}M_{ij14}\right|,\text{log}\left(\left|M_{ij1}M_{ij2}M_{ij6}\right|\right.\left.\left.+\left|M_{ij5}M_{ij7}M_{ij8}+0.01M_{ij15}\right|\right)\right\}$. The risk scores are generated by $r_{ij}=X_{ij}^{T}\beta -4+b_{i}$, and $\beta ={\left(\beta_{1},\beta_{2},\beta_{3},\beta_{4}\right)}^{T}=(1,1,1,1{)}^{T}$. The censoring times are generated from Uniform(0, 15) with around 70% of the right censoring rate.

Tables 1 and 2 show the results under different frailty variances for scenarios 1 and 2, respectively. The proposed method and Deepsurv are fitted under a two-layer neural network with (64, 64) hidden nodes. Following Kvamme et al. (2019), hyperparameters in the simulation study and real data analysis are selected through a random search on the validation set over the relevant parameters in Table 5 in the Appendix. We train the model for 100 epochs with a batch size of 128, a dropout rate of 0.2, and a weight decay of 0.001. The learning rate is dynamically adjusted based on the optimal rate identified by the learning rate finder. In terms of training stability, we implement early stopping with a patience threshold to halt training if no improvement is observed, ensuring stability across different runs. We ensure model stability by setting early stopping criteria and observing training consistency across multiple runs. These settings contribute to reliable training performance while maintaining computational efficiency.

Under both scenarios, our method yields the best AUC among all the methods when the random effect variance is large, e.g., θ=2.5or3.5. This demonstrates the advantage of our model in capturing the heterogeneity across clusters and characterizing the complex nonlinear and interactive covariate effects simultaneously. Specifically, the distinction between our proposed method and Deepsurv grows more pronounced as the value of θ escalates, primarily because our method integrates a frailty term to account for cluster effects. In Tables 1 and 2, we have reported the standard deviations for the C-index. The proposed method and the DeepSurv method exhibit consistently low standard deviations under both scenarios, demonstrating the stable performance of these deep learning approaches.

We have included the average computational time for each method in the simulation studies to provide insights into computational efficiency for potential users. As shown in Table 3, the proposed method and DeepSurv require more time compared to standard Cox models, but their computational times are similar to the Cox model with fixed clustering effects and are less than the Cox frailty model with interactions. This result demonstrates that while the proposed and DeepSurv methods involve greater computational costs than simpler Cox models, they are comparable to more complex Cox models with frailty terms.

## 4 Kidney Transplant Data Analysis

We compare the accuracy of the proposed method with six other competing methods in predicting the time-to-graft-failure or death after kidney transplantation using the national kidney transplant registry data obtained from U.S. Organ Procurement and Transplantation Network (https://optn.transplant.hrsa.gov/data/). OPTN aims to improve the U.S. donation and transplantation system so that more life-saving organs are available for transplant. Following Liu et al. (2023), our study focuses on a cohort of 8,378 adult individuals (those aged 18 or older) who underwent kidney transplant between January 1st, 2007, and December 31st, 2007. These individuals were treated at 154 medical facilities, with the number of patients treated in each facility ranging from 20 to 205. Out of the 8,378 patients, 2,280 encountered either death or graft failure after the kidney transplant. The remaining patients were censored after a five-year post-transplant follow up, with a censoring rate of 72.78%.

In the analysis, we include 15 baseline factors: time on end-stage renal disease (ESRD), donor age, donor gender (male = 1, female = 0), donor body mass index (BMI), donor race (reference: white), donor history of hypertension (yes = 1, no = 0), donor meeting expanded criteria (yes = 1, no = 0), recipient gender (male = 1, female = 0), recipient race (reference: white), recipient insulin-dependent diabetes (yes = 1, no = 0), recipient non-insulin dependent diabetes (yes = 1, no = 0), recipient age at transplant, recipient BMI, whether the recipient received a previous kidney transplant (yes = 1, no = 0), recipient total cold ischemia time.

We apply our proposed method to predict the post-transplant graft failure or death for patients within each facility, which is regarded as a cluster. The goal of our analysis is to use the time-to-graft-failure or death of subjects in the training dataset to predict that of the subjects in the test dataset from the same facility. The ReLU activation function is selected for its faster convergence rate and better performance (Maas et al. (2013)).

Table 4 reports the C-indexes in five-fold cross-validation (CV) for performance comparison. Within each facility, we randomly assign 80% of its patients as the training set and the remaining 20% as the testing set. The proposed method has the highest average C-index among all the methods, indicating the advantages of incorporating non-linearity, interaction, and clustering effects in the risk function. Accurate predictions within the same facility are pivotal for identifying patients with a high risk of graft failure or subsequent death post-transplantation. This identification aids in averting excessive treatments or suboptimal resource distribution.

To assess the calibration ability of the models, we also obtain the time-dependent Brier score (Gerds and Schumacher (2006), Sun et al. (2020)). The time-dependent Brier score is an extension of the Brier score, which takes into account the predicted survival probabilities at different time points and compares them to the actual survival probabilities over time. The time-dependent Brier score measures the mean square error between the observed status $Y_{i}(t)=I\left(U_{i}>t\right)$ and the predicted survival probability $S\left(t|X_{i},Z_{i}\right)$ for subject i at time t. The Brier score, ranging between 0 and 1, reflects the accuracy of probabilistic predictions. A score of 0 signifies perfect prediction, where the predicted probabilities align precisely with the actual outcomes. A lower Brier score indicates enhanced calibration performance and greater accuracy of the model’s probabilistic predictions at a given time point.

We estimate the time-dependent Brier score on the test dataset by the inverse probability weighting method (Sun et al. (2020)):

$$\hat{BS}\left(t,\overset{ˆ}{S}\right)=\frac{1}{M}\sum_{i\in D_{M}}{\overset{ˆ}{W}}_{i}\left(t\right){\left\{Y_{i}\left(t\right)-\overset{ˆ}{S}\left(t|X_{i},Z_{i}\right)\right\}}^{2},$$

where $D_{M}$ is the test dataset with size M, and ${\overset{ˆ}{W}}_{i}(t)=\frac{\left(1-Y_{i}(t)\right)\delta_{i}}{\overset{ˆ}{G}\left(Y_{i}-\right)}+\frac{Y_{i}(t)}{\overset{ˆ}{G}(t)}$ is the inverse probability of censoring weights with $\overset{ˆ}{G}(t)=\overset{ˆ}{P}(C>t)$.

**Figure 2:**
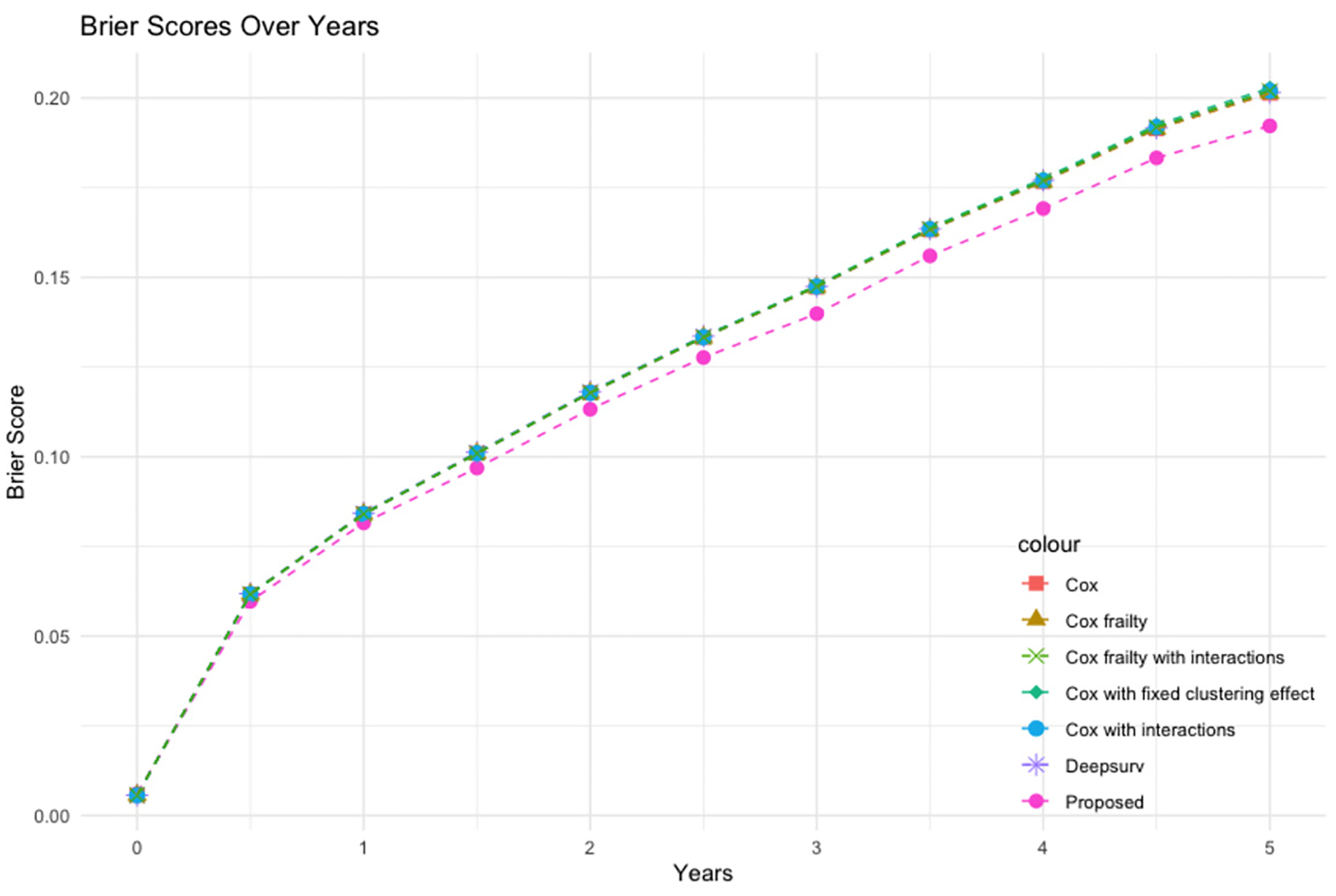
The average time-dependent Brier scores for the cross-validation datasets from seven prediction models (Proposed, Deepsurv, Cox frailty, Cox frailty with interactions, Cox, Cox with interactions, Cox with fixed clustering effects).

Similar to Sun et al. (2020), we report time-dependent Brier scores at different time points. Figure 2 presents the average time-dependent Brier scores on the five-fold cross-validation datasets under each prediction model. As shown in Figure 2, the Brier scores from our model are consistently lower than all the other models across all time points, indicating its superior performance in both discrimination and calibration. For a comprehensive view of the Brier scores across all seven methods at various time points, refer to Table 6 in the Appendix.

## 5 Discussion

We propose a neural network for correlated survival outcomes. The proposed method extends the classical neural network framework to include a random effect (frailty) accounting for within-cluster correlation. The model uses a feed-forward neural network for nonlinear and interactive fixed effects and estimates random effects in the last layer of the neural network to avoid iterative computation. The neural network is trained over a loss function derived from the penalized partial likelihood with a Laplace approximation. Through both simulation studies and realworld data evaluations, the advantages of our method are evident over conventional survival regression techniques and Deepsurv. In summary, our method stands out as an effective instrument for predicting correlated survival outcomes, particularly in modeling intricate covariate impacts.

There are several future directions in this research. First, while this study focuses on correlated survival outcomes in clusters, exploring methods for recurrent event data represents a captivating progression. This would delve deeper into another aspect of correlated survival outcomes (Cook et al. (2007)). Second, it would be of interest to develop deep learning prediction methods for correlated survival outcomes using e.g., the additive hazards model (Aalen (1989)) or the linear transformation model (Fine et al. (1998)). Third, this paper primarily focuses on predicting correlated survival endpoints, treating the random effects as nuisance parameters. In contrast, in provider profiling, the individual effect $b_{i}$ is of central importance (Normand and Shahian (2007); He et al. (2013); Liu et al. (2023)). Extending our method to address provider profiling represents a promising direction for future research. Finally, we only consider time-independent covariates (at baseline) for predicting time to event. It is of importance to consider longitudinal biomarkers for dynamic prediction in the joint model and landmark model frameworks (Rizopoulos et al. (2017); Tanner et al. (2021); Lin and Luo (2022)).

## Supplementary Material

To enhance reproducibility, we make all computer programs and sample data used for implementations available on https://github.com/rivenzhou/deep_learning_clustered. Restrictions apply to the availability of the real data, which were used under license for this study.

## Figures and Tables

**Table 1:** C-index for 100 simulated test datasets in scenario 1 with simple quadratic nonlinear fixed effects (standard deviations in brackets).

Frailty Variance	Prop.	DS	CF-Lin	CF-Int	Cox	Cox-Int	Cox-FC	TM
0	72.06 (0.10)	79.07 *(0.08)*	50.04 (0.01)	50.54 (0.01)	50.05 (0.01)	50.55 (0.01)	50.01 (0.01)	83.92 (0.01)
1.5	78.05 (0.09)	78.21 (0.05)	50.06 (0.01)	50.26 (0.01)	50.05 (0.01)	50.18 (0.01)	67.96 (0.01)	87.24 (0.01)
2.5	76.56 (0.09)	76.35 (0.04)	50.08 *(0.02)*	50.20 (0.01)	50.06 (0.01)	50.14 (0.01)	72.86 (0.01)	88.39 (0.01)
3.5	78.45 (0.04)	72.88 (0.05)	50.10 (0.01)	50.17 (0.01)	50.05 (0.01)	50.05 (0.01)	76.13 (0.01)	89.31 (0.01)

Abbreviations: Prop. (Proposed model), DS (DeepSurv), CF-Lin (Cox frailty with linear effects), CF-Int (Cox frailty with interactions), Cox (Cox proportional hazards model), Cox-Int (Cox model with interactions), Cox-FC (Cox model with fixed clustering effects), TM (True model).

**Table 2:** C-index for 100 simulated test datasets in scenario 2 with complex nonlinear fixed effects (standard deviations in brackets).

Frailty Variance	Prop.	DS	CF-Lin	CF-Int	Cox	Cox-Int	Cox-FC	TM
0	64.42 (0.03)	66.90 (0.03)	73.82 (0.24)	71.89 (0.30)	73.82 (0.24)	71.93 (0.40)	70.66 (0.25)	77.29 (0.39)
1.5	71.03 (0.05)	62.96 (0.03)	68.07 (0.25)	69.11 (0.27)	68.51 (0.25)	68.36 (0.30)	67.28 (0.27)	79.12 (0.38)
2.5	74.43 (0.05)	60.85 (0.04)	69.67 (0.24)	63.92 (0.32)	70.61 (0.24)	67.47 (0.30)	62.75 (0.30)	80.99 (0.37)
3.5	74.67 (0.08)	59.61 (0.07)	69.35 (0.24)	63.78 (0.30)	70.77 (0.24)	67.35 (0.30)	62.39 (0.30)	75.98 (0.41)

Abbreviations: Prop. (Proposed model), DS (DeepSurv), CF-Lin (Cox frailty with linear effects), CF-Int (Cox frailty with interactions), Cox (Cox proportional hazards model), Cox-Int (Cox model with interactions), Cox-FC (Cox model with fixed clustering effects), TM (True model).

**Table 3:** Average computational time in seconds for simulation studies.

Prop.	DS	CF-Lin	CF-Int	Cox	Cox-Int	Cox-FC	TM
3.05	3.67	0.44	3.90	0.28	0.53	2.69	0.39

Abbreviations: Prop. (Proposed model), DS (DeepSurv), CF-Lin (Cox frailty with linear effects), CF-Int (Cox frailty with interactions), Cox (Cox proportional hazards model), Cox-Int (Cox model with interactions), Cox-FC (Cox model with fixed clustering effect), TM (True model).

**Table 4:** C-index on the kidney transplant data.

Prop.	DS	CF-Lin	CF-Int	Cox	Cox-Int	Cox-FC
59.24	55.18	53.46	55.22	53.48	55.23	53.46

Abbreviations: Prop. (Proposed model), DS (DeepSurv), CF-Lin (Cox frailty with linear effects), CF-Int (Cox frailty with interactions), Cox (Cox proportional hazards model), Cox-Int (Cox model with interactions), Cox-FC (Cox model with fixed clustering effects).

## A Appendix

**Table 5:** Hyperparameter search space for simulation study and real data analysis.

Hyperparameter	Values
Layers	(1, 2, 3}
Nodes per layer	{16, 32, 64, 128}
Weight decay	(0.001, 0.01, 0.1, 0.2, 0.4}
Batch size	(64, 128, 256}
Dropout rate	(0.1, 0.2}
Epoch	(100,200}

**Table 6:** Time-dependent Brier scores on the kidney transplant data at years 1, 2, 3, 4, and 5 after the kidney transplantation.

Time	Prop.	DS	CF-Lin	CF-Int	Cox	Cox-Int	Cox-FC
1	0.0815	0.0843	0.0840	0.0841	0.0840	0.0840	0.0841
2	0.1132	0.1181	0.1178	0.1178	0.1177	0.1178	0.1180
3	0.1398	0.1475	0.1473	0.1475	0.1473	0.1475	0.1477
4	0.1691	0.1770	0.1770	0.1770	0.1767	0.1770	0.1775
5	0.1922	0.2015	0.2013	0.2020	0.2013	0.2020	0.2028

Abbreviations: Prop. (Proposed model), DS (DeepSurv), CF-Lin (Cox frailty with linear effects), CF-Int (Cox frailty with interactions), Cox (Cox proportional hazards model), Cox-Int (Cox model with interactions), Cox-FC (Cox model with fixed clustering effects).
